# Evaluation of surface tensions and root-dentin surface contact angles of different endodontic irrigation solutions

**DOI:** 10.1186/s12903-024-04453-w

**Published:** 2024-06-12

**Authors:** Hatice Buyukozer Ozkan, Arslan Terlemez, Ahmet Burcin Batibay, Hilal Erdogan, Funda Kont Cobankara

**Affiliations:** 1 Faculty of Dentistry, Department of Endodontics, Alanya Alaaddin Keykubat University, Konaklı Mah. Mustafa Kemal Blv. No:82 Alanya, Antalya, Türkiye; 2 Faculty of Dentistry, Department of Endodontics, Necmettin Erbakan University, Konya, Türkiye; 3 Faculty of Engineering, Metallurgical and Materials Engineering, Necmettin Erbakan University, Konya, Türkiye; 4 Faculty of Dentistry, Department of Endodontics, Nevsehir Haci Bektas Veli University, Nevsehir, Türkiye; 5 Faculty of Dentistry, Department of Endodontics, Selcuk University, Konya, Türkiye

**Keywords:** Contact angle, Surface tension, Irrigation solution, Root canal dentin

## Abstract

**Background:**

Surface tension and contact angle properties, which play a crucial role in determining the effectiveness of irrigation solutions in penetrating dentin surfaces and dentin tubules, are highly important for the development of new irrigation solutions and their preferences. The aim of the current study was to compare the surface tension and contact angle properties of different irrigation solutions used in endodontics, both on the dentin surface and within dentin tubules.

**Methods:**

In this study, the contact angles and surface tensions of 5.25% sodium hypochlorite (NaOCl), 17% ethylenediaminetetraacetic acid (EDTA), 2% chlorhexidine (CHX), 5% boric acid (BA), 0.02% hypochlorous acid (HOCl), 0.2% chlorine dioxide (ClO_2_), Biopure MTAD, QMix solutions, and distilled water (control group) were measured. Measurements were conducted using a goniometer device (Attension Theta Lite Tensiometer, Biolin Scientific, USA), employing the sessile drop method for contact angle measurements on pre-prepared dentin surfaces, and the pendant drop method for surface tension.

**Results:**

Contact angle measurements revealed no statistically significant differences between the contact angle values of MTAD, ClO_2_, and CHX or between NaOCl, QMix, BA, and HOCl (*p* > 0.05). However, EDTA exhibited a significantly greater contact angle than did MTAD, ClO_2_, CHX, NaOCl, QMix, BA, and HOCl (*p* < 0.05). Furthermore, the contact angle of dentin with distilled water was greater than that with all other solutions tested (*p* < 0.05). Surface tension measurements revealed that the surface tension values of QMix and MTAD were statistically similar (*p* > 0.05). CHX exhibited lower surface tension than distilled water and HOCl (*p* < 0.05), and it also had lower surface tension than ClO_2_, NaOCl, and BA (*p* < 0.05). Additionally, the surface tension of the samples treated with EDTA was greater than that of all other solutions tested (*p* < 0.05).

**Conclusion:**

The direct linear relationship between the surface tension of liquids and contact angles on different surfaces may not always hold true, and these values should be considered independently for each solution on various surfaces. Considering the contact angles and surface tension properties of irrigation solutions with root canal dentin, it can be suggested for clinical use that ClO_2_ could be recommended over NaOCl, and similarly, BA could be recommended over EDTA.

## Background

Effective disinfection of the root canal system largely depends on the ability of irrigation solutions to contact dentin walls and debris, as well as their capacity to penetrate dentin surfaces and dentin tubules [1]. Understanding the mechanisms of wetting and spreading can significantly contribute to the understanding of the effectiveness of irrigation solutions. Therefore, knowing the contact angle and surface tension properties is highly important [2]. It is desirable that the solutions to be used have low contact angles and low surface tension properties, which are important requirements.

The angle formed by a liquid in contact with a solid surface is called the contact angle. The contact angle provides information about the wettability of a liquid on a solid material [3]. As the contact angle decreases, the liquid can more easily enter capillary pores and wet the solid material. The ability of irrigation solutions to penetrate deeply into dentin surfaces and dentin tubules is also related to the surface tension of the liquid [4]. Surface tension is defined as the force between molecules that creates a tendency for a liquid to minimize its surface area [5]. This force tends to limit the ability of a liquid to penetrate into a capillary tube. Therefore, irrigation solutions with low surface tension are interpreted to have greater wetting effectiveness. The wetting ability of the solution can affect both the main and lateral canals as well as the penetration capability into dentin tubules [6, 7]. Additionally, it can enhance protein-dissolving capacity and provide better disinfection and antimicrobial efficacy in areas of the root canal system that are inaccessible with biomechanical preparation [6].

Sodium hypochlorite (NaOCl) is currently the most widely used irrigation solution, especially due to its solvent effect on pulp tissue, high antibacterial activity, and lubricating properties [8]; however, its cytotoxicity at high concentrations and inadequacy in removing the smear layer alone have led to research into alternative solutions [8]. For this purpose, solutions such as boric acid (BA), chlorine dioxide (ClO_2_), and hypochlorous acid (HOCl) continue to be evaluated for their potential as irrigation solutions due to their different properties [8–10]. Additionally, due to its inadequacy in removing the smear layer alone, it is necessary to use two or more irrigation solutions sequentially. Therefore, combined irrigation solutions such as QMix (Dentsply Tulsa, Maillefer, Ballaigues, Switzerland) and MTAD (Dentsply, Tulsa Dental, Tulsa, OK, USA) have also been introduced to the market after irrigation with NaOCl [11].

According to our current knowledge, various irrigation solutions have been measured for their surface tensions and contact angles with dentin surfaces in the literature. However, there is no research that simultaneously compares both the surface tensions and contact angles of the solutions used in the present study. Therefore, the null hypothesis of our study is that there is no difference in the contact angles of irrigation solutions with dentin, and the second null hypothesis is that there is no difference in the surface tensions of irrigation solutions. We believe that our study, which we think will contribute to the literature, will provide valuable insights.

## Methods

Ethical approval for the study was obtained from the Ethics Committee for Non-Drug and Non-Medical Device Research of the Necmettin Erbakan University Faculty of Dentistry (Decision #: 2021/01–06). The solutions to be measured for surface tension and contact angle are shown in Table 1.

**Table 1:** The concentrations, manufacturer companies, and pH values of the solutions used in the study

Solution	pH	The manufacturer company
5.25%NaOCl	12.8	Endosolve HP, Imicryl, Konya, Türkiye
17%EDTA	12.1	Imicryl, Konya, Türkiye
2% CHX	7	Ceraxidin-C, Imicryl, Konya, Türkiye
5% BA	4.8	Sigma Aldrich, Darmstadt, Germany
0.02% HOCl	6.7	Crystalin, Natural Health Products-NHP,Türkiye
0.2% ClO_2_	1.2	AgrOx® Ankara, Türkiye
Biopure MTAD	2.15	Tulsa Dental Specialties Company,OK,USA
QMix	7.5–8.0	Tulsa Dental Specialties Company,OK,USA
Distilled water	7	

### Sample preparation

In the study, a power analysis resulted in 12 samples in each group with 85% power; a total of 54 human central incisors, extracted due to periodontal reasons without any fractures or caries, were used. The crowns of the teeth were removed with a diamond bur under water cooling, and the remaining root portions were vertically cut into two equal parts along the root length at 300 rpm using a precision cutting machine (Buehler Precision Saw, USA) under water cooling. Subsequently, each root half was embedded horizontally in self-curing acrylic resin (Ivoclar Vivadent, Zurich, Switzerland) so that only the root dentin surfaces remained exposed, and the exposed root dentin surfaces were polished with #180-grit sandpaper under distilled water (Milli-Q; Millipore, Billerica, MA) using a Tronic Ecopol 200 Polisher (Metcon, Bursa, Turkey) to obtain flat and wide dentin surfaces. The other surface of the acrylic resin was also sanded to obtain a perfectly parallel surface.

### Contact angle measurement

**Fig. 1:**
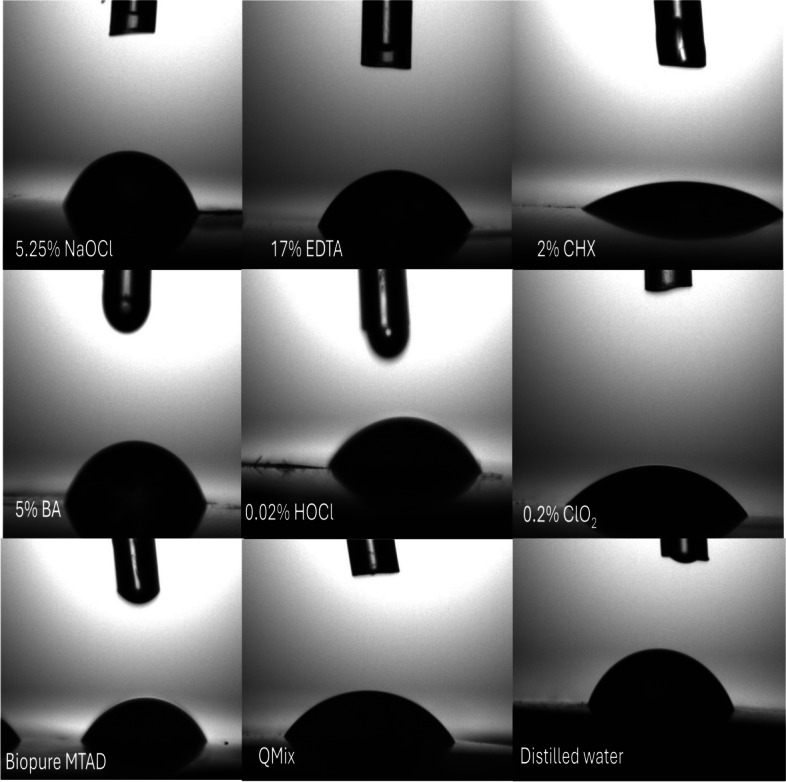
These pictures are images that captured during the measurement of contact angles of tested irrigation solutions on dentin surfaces

The obtained samples were dried with compressed air at 5.5 bar pressure for 2 s and contact angle measurements were performed using a camera-based contact angle measurement device, the goniometer, with the sessile drop method at 20 °C. (Attension Theta Lite Tensiometer, Biolin Scientific, USA) [12–14]. The root sample embedded in acrylic resin with a flat lower surface was fixed onto the device plate using adhesive wax and positioned so that the measurement surface was parallel to the plate. After positioning the mid-third of the root surface parallel to the plate, the measurement was initiated by dropping the test solution onto the mid-third surface of the root dentin using a micropipette. Images were captured using a high-resolution camera on the device (with the capacity to take a different number of photos per second) for 10 s from the moment of dropping, and then transferred to a computer to calculate the contact angles of the solutions. Figure 1 shows an example of photographs of contact angle measurements for all solutions.

### Surface tension measurement

**Fig. 2:**
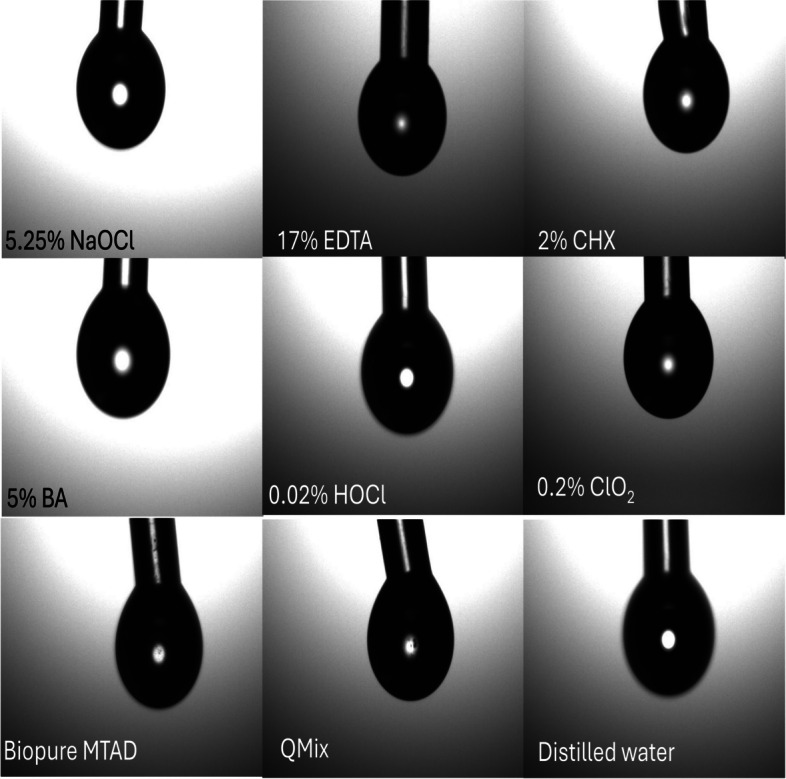
These pictures are images that captured during the measurement of surface tension of tested irrigation solutions

Surface tension measurements of the solutions used in the study were performed using a goniometer (Attension Theta Lite Tensiometer, Biolin Scientific, USA) with the pendant drop method [15, 16]. In this method, the device records drop images and automatically analyzes the drop shape. For analysis, the device was set to have a single drop volume of 5 μL each time, with a total analysis time of 10 s per drop, and recordings were taken every 0.1 s. Microscopy images obtained with the device were then converted to surface tension values in mN/m units using internal software. To increase measurement accuracy, three different measurements were taken from each solution, and the average surface tension value was obtained. The tests were carried out at a temperature of 20 °C. Figure 2 shows an example of photographs of surface tension measurements for all solutions.

### Statistical analysis

The normality of the parameters was evaluated using the Shapiro‒Wilk and Kolmogorov‒Smirnov tests during the assessment of the study data. One-way analysis of variance (ANOVA) was used for comparisons between groups of parameters. The data were analyzed using the IBM SPSS Statistics V22 (IBM SPSS, USA) statistical package program. Analysis results were considered at a 95% confidence level, and p values less than 0.05 were considered statistically significant.

## Results

The average contact angle measurements and statistical data are shown in Table 2. The contact angles are arranged from smallest to largest as follows: Biopure MTAD ≤ CHX ≤ ClO_2_ < NaOCl ≤ QMix ≤ BA ≤ HOCl < EDTA < distilled water. There is no statistically significant difference between Biopure MTAD, CHX, and ClO_2_ (*p* > 0.05). Similarly, there is no statistically significant difference between the contact angle values of NaOCl, QMix, BA, and HOCl (*p* > 0.05). The contact angle of EDTA is statistically significantly higher than that of Biopure MTAD, ClO_2_, CHX, NaOCl, QMix, BA, and HOCl (*p* > 0.05). The contact angle of distilled water with dentin is statistically significantly higher than that of all other tested solutions (*p* < 0.05).

**Table 2:** Mean contact angle measurement values and statistical evaluation results (*n* = 12)

**Solutions**	Mean ± SD*	**95% Confidence Interval for Mean**	
	**(°)**	**Lower Bound**	**Upper Bound**
**5.25%NaOCl**	48.81 ± 6.18^**b**^	44.88	52.74
**17%EDTA**	65.83 ± 6.33^**c**^	61.81	69.85
**2% CHX**	39.14 ± 3.72^**a**^	36.78	41.50
**5% BA**	53.81 ± 8.43^**b**^	48.46	59.17
**0.02% HOCl**	54.24 ± 3.95^**b**^	51.73	56.74
**% 0.2 ClO**_**2**_	39.48 ± 8.28^**a**^	34.22	44.74
**Biopure MTAD**	38.48 ± 4.68^**a**^	35.51	41.45
**QMix**	49.98 ± 3.24^**b**^	47.92	52.03
**Distilled water**	75.92 ± 3.28^**d**^	73.83	78.00

^*^Statistically significant differences exist between different letters in the same column (*p* < 0.05)

The average surface tension values ​​of the tested solutions and their statistical analysis are shown in Table 3. The surface tension of the tested solutions is arranged from smallest to largest as follows: QMix ≤ Biopure MTAD < CHX < distilled water ≤ HOCl < ClO_2_ < NaOCl ≤ BA < EDTA. The surface tension values ​​of QMix and Biopure MTAD are statistically similar (*p* > 0.05). Distilled water and HOCl exhibit statistically similar surface tension properties (*p* > 0.05), and both have lower surface tension than ClO_2_ (*p* > 0.05). NaOCl and BA show similar surface tension properties (*p* > 0.05); however, EDTA's surface tension is statistically significantly higher than that of all other tested solutions (*p* > 0.05).

**Table 3:** Surface tension values (mN/m) of tested solutions at 20 °C (*n* = 12)

**Solutions**	Mean ± SD*	**95% Confidence Interval for Mean**	
	**(mN/m)**	**Lower Bound**	**Upper Bound**
**5.25%NaOCl**	81.77 ± 0.73^**e**^	81.30	82.24
**17%EDTA**	98.85 ± 1.15^**f**^	98.11	99.59
**2% CHX**	61.72 ± 0.98^**b**^	61.1	62.35
**5% BA**	82.06 ± 2.42^**e**^	80.52	83.59
**0.02% HOCl**	72.9 ± 1.03^**c**^	72.24	73.55
**0.2%ClO**_**2**_	77.08 ± 1.64^**d**^	76.03	78.12
**Biopure MTAD**	40.94 ± 0.82^**a**^	40.42	41.46
**QMix**	39.40 ± 0.61^**a**^	39.01	39.79
**Distilled water**	72.8 ± 0^**c**^	72.8	72.8

^*^Different letters for each column indicate statistical significance among groups (*p* < 0.05)

## Discussion

Solid surface free energy and liquid surface tension are the main factors that affect the wetting of a solid surface. The contact angle provides information about the wettability of a material and the surface energy of a solid surface. When the contact angle is > 90°, the liquid will flow away from the surface, while when the contact angle is < 90°, the liquid will wet the solid material, and when the contact angle is zero, the surface will be completely wet [17]. Therefore, knowing the contact angles of the solutions used in root canal irrigation, especially with respect to the root canal dentin, is a crucial criterion in determining their clinical effectiveness. Hence, in our current study, the contact angle measurements of the tested solutions were performed on the surfaces of root canal dentin. Although there are few studies in the literature evaluating the contact angle on the dentin surface, these studies used single-rooted human teeth [18–20]. In order to make comparison and meta-analysis more effective for past and future studies, human single-rooted teeth were preferred in our study. In addition, wide and straight canals in this type of single-rooted teeth enable irrigation solution application research and activation efficiency research to be carried out in laboratory environments in a more standardized manner [8, 21, 22].

The roughness of the solid surface is also an important factor that affects the rate of liquid absorption [17]. Polishing the surface alters roughness, thus affecting the resulting contact angle [23]. Studies have pointed out the significance of polishing the dentin surface before contact angle analyses [18]. For this reason, each sample surface was polished with #180-grit sandpaper under distilled water using a Tronic Ecopol 200 Polisher (Metcon, Bursa, Turkey) to ensure standardization before contact angle measurements and smooth dentin surfaces were obtained. However, the samples were not additionally tested for surface roughness before the measurements.

The behavior of irrigation solutions on smooth dentin surfaces can provide information regarding their ability to reach complex recesses in the root canals. Factors such as pressure applied during irrigation, temperature changes, root curvature, and tubule anatomy can affect the surface tension and contact angle of the irrigant [24], but the purpose of polishing dentin surfaces in this study was to standardize the calculation of the contact angle and assess the wettability of the fluids used in the experiment by reducing other factors. Thus, future studies involving staining or fluorescence techniques are needed to measure the extent to which irrigation solutions can reach the recesses within the root canal.

The most commonly used method for determining the contact angles of liquids is the sessile drop method, where a goniometer device is used for measurements [17]. Alongside measuring contact angles, surface tension can also be determined using a goniometer device [17]. Since surface tension measurements can be conducted using the same device and setup as contact angle measurements, in our study, liquid surface tension was measured using the pendant drop method. Since contact angle measurements are independent of the volume of the drop on the sample [17], the volume of the drops applied to the sample surfaces was not specifically mentioned in our study.

Based on the statistical data, the average surface tension values for the tested solutions in the current study can be ranked as follows: QMix = Biopure MTAD < CHX < distilled water = HOCl < ClO_2_ < NaOCl = BA < EDTA. Similarly, the average contact angle values of the same solutions on dentin surfaces can be ranked as Biopure MTAD = ClO_2_ = CHX < NaOCl = QMix = BA = HOCl < EDTA < distilled water. Considering these data, both null hypotheses in our study have been partially rejected.

Literature indicates various methods for surface tension measurement, including the DuNouy ring method [5],Wilhelmy plate method [25, 26], capillary method [4], or pendant drop techniques [16]. In our current study, the pendant drop technique was utilized for surface tension measurements. While these methods are fundamentally based on similar concepts, the surface tension levels measured using different methods for the same solutions can vary significantly [16, 25, 27, 28].

Göktürk et al. [27] conducted an in vitro study using the DuNouy ring method to examine various irrigation solutions, measuring the surface tension values of 17% EDTA and 5% NaOCl solutions as 59.26 and 70.36, respectively, at 22–23 °C. In their research, the surface tension of NaOCl was found to be statistically significantly higher than that of EDTA, contradicting the findings of our study. Giardino et al. [25], measured the surface tension values of Biopure MTAD, 17% EDTA, and 5% NaOCl solutions as 34.5, 46.8, and 49, respectively, at 22 °C using the Wilhemny plate method. Ulusoy et al. [28], using the sessile drop method, measured the surface tension of 17% EDTA and 5.25% NaOCl solutions at room temperature (20–22 °C) as 67.64 and 56.98, respectively, and found them to be statistically similar. Yılmaz et al. [16] used the pendant drop technique to measure the surface tension of 17% EDTA at different temperatures and pH levels. They determined this value to be 70 at 22 °C and pH 7.5. The same researchers also suggested that different preparation conditions, such as pH or temperature of EDTA, could lead to different surface tension levels, and the surface tension of the solution could also change depending on the shelf life of ready-made solutions [16]. In our study, the surface tension of 17% EDTA solution at pH 12.1 was determined to be 98.85 at 20 °C, which was statistically significantly higher than that of 5.25% NaOCl solution. In the study by Yılmaz et al. [16], it was reported that when prepared at room temperature, the EDTA solution showed similar surface tension levels at pH 5.5 and 10.5, while it exhibited significantly lower levels at pH 7.5. The same study also noted that increasing the temperature significantly reduced the surface tension of the EDTA solution at pH 5.5 and 7.5 but did not cause a significant change at pH 10.5. The higher surface tension measurement value in our study compared to other studies may be attributed to the measurements being conducted at 20 °C, differences in pH values of EDTA, and/or differences in the methodology.

Boric acid (BA) is a notable irrigation solution with potential diverse applications in endodontics, as it not only exhibits anti-inflammatory effects but also removes the smear layer, exposes collagen fibrils, and opens dentinal tubules due to these properties [29]. Sakhipgareev et al. [30] measured the surface tension of 5% BA using the duNouy ring method at 26 °C, obtaining a value of 71. They noted variations in surface tension, with values of 69 at 40 °C and 64 at 70 °C, indicating a dependency on concentration and temperature. In our current study, the surface tension of BA was measured as 82.06, potentially influenced by the temperature of 20 °C and the use of the pendant drop method in our experiment, explaining the difference.

Lee et al. [31] examined the contact angle of BA on solid surfaces, reporting a decrease in angle with increasing concentration. They noted that the contact angle of BA on solid surfaces ranged from 40° to 36.9° for concentrations between 0.2% and 0.6%. On a solid surface, the shape of the droplet and the contact angle of the solution are also dependent on the surface free energy and topography of the solid [17]. To achieve accurate contact angle measurements, it is essential for the solid surface to be both clean and smooth, as well as hard. However, root canal dentin surfaces do not possess the required level of smoothness even after the cleaning and shaping process. The concentration of BA used in our investigation was 5%, and we assessed the contact angle on polished dentin surfaces to be 53.81°. The discrepancy may be attributed to variations in the concentration that was used in prior investigations and the method of measuring contact angles on dentin surfaces. However, the lack of adequate evidence in the literature required further research to arrive at a conclusive assessment.

The present study found that EDTA and BA exhibited higher surface tension compared to distilled water; however, their contact angles were statistically significantly lower than those of distilled water. While there is literature available on contact angle measurements of various solutions, there are relatively few studies specifically evaluating contact angles on direct dentin surfaces [18–20]. There has not been a study specifically evaluating the contact angle of 17% EDTA and 5% BA on dentin surfaces. EDTA is a chelating agent that reacts with calcium ions in dentin to form soluble calcium chelates. [32]. It is also known that BA removes the smear layer [29]. It is also known that the contact angle is affected by chemical reactions occurring at the interface [33]. This reaction could explain why EDTA and BA exhibit a lower contact angle compared to distilled water in our study; however, further research is required to investigate this matter.

In the literature, the surface tension of NaOCl at various concentrations has been measured using different methods [34]. Leonardo et al. [34] measured the surface tension of NaOCl using the DuNouy ring method at 25 °C and found it to be 66.41. Spano et al. [35] measured the surface tension of 5% NaOCl using a tensiometer at 22 ± 2 °C and found it to be 77.3. Sevimay et al. [24] conducted surface tension measurements of NaOCl at various temperatures and concentrations using the du Nouy ring method. Their findings indicated that elevated temperatures correlated with a reduction in surface tension, whereas higher concentrations were associated with an increase in surface tension. Additionally, they determined the surface tension of 5.25% NaOCl at 21 °C to be 67.13. However, our study recorded this value as 81.77 at 20 °C.

BioPure MTAD is a final irrigation solution composed of doxycycline, citric acid, and Tween 80. With a pH value of 7.0, it is considered a biologically acceptable irrigant and is claimed to be usable as a single solution for final irrigation purposes [36]. Tween 80 (polyoxyethylene sorbitan monooleate) is a nonionic surfactant and detergent [36]. Çiçek et al. [37] measured the surface tensions of various irrigation solutions using the Traube stalagmometer with the drop method. They determined the surface tension values of BioPure MTAD, 2% CHX, and 5.25% NaOCl to be 48.53, 60.66, and 84.93, respectively, which is consistent with the findings of our study. Giardino et al. [26] measured the surface tension of Biopure MTAD and Qmix solutions using the Wilhelmy plate method, determining them to be 34.54 and 36.43, respectively. They found no statistically significant difference between these two solutions, which is consistent with the results of our study. Altundasar et al. [38] measured the surface tension of Biopure MTAD and 17% EDTA solutions at room temperature using the pendant drop method, determining them to be 37.45 and 79.90, respectively. Their findings align with the results of our study. While there are studies evaluating the surface tension of Biopure MTAD in the literature, there is no study measuring the contact angle on dentin surfaces. In this regard, our research contributes to the literature.

QMix, introduced to the market in 2011, serves as a final irrigation solution after NaOCl for the simultaneous removal of the smear layer and dentin disinfection. It contains EDTA, CHX, and a detergent (cetrimide), with a pH slightly above neutral [26]. In their in vitro study, Giardino et al. [26] examined the surface tension of QMix using the Wilhelmy plate method and measured it as 36.43. Our current research revealed a surface tension of 39.40 for QMix, and a contact angle of 49.98 on dentin surfaces. To our knowledge, there has been no prior investigation into the contact angle of QMix with root canal dentin, making our study the first to provide data in this regard. Consequently, further research is warranted to expand on this topic.

CHX is a broad-spectrum antimicrobial agent highly effective against gram-positive and gram-negative bacteria as well as yeasts. Because it is cationic, CHX can breakdown the outer layers of cells, letting things pass through them, and its electrostatic layers can stick to the outer walls of bacteria [39]. Sevimay et al. [24] measured the surface tension of 5.25% NaOCl and 2% CHX using the Du Nouy method at 21 °C, reporting that 2% CHX exhibited a statistically significant lower surface tension compared to 5.25% NaOCl. Additionally, according to the same study data, the researchers also reported a decrease in surface tension with increasing temperature. Abbaszadegan et al. [40] measured the surface tension of 5.25% NaOCl, 2% CHX, and 17% EDTA using the Wilhelmy plate method at 25 °C. They reported that 2% CHX exhibited a statistically significant lower surface tension compared to the other two solutions. These findings are consistent with our study results. Estrela et al. [41] calculated the surface tension value of 2% CHX using a tensiometer at 25 °C to be 55.5. However, our current study's results determined this value to be 61.72.

Basrani et al. [20] determined the contact angle of CHX on the dentin surface to be 28.4. Thirunarayanan and Hegde [19] calculated the contact angle of 2% CHX on the coronal root dentin surface to be 21.54. We determined this value to be 39.14 in our study. Natural variations in the root dentin surfaces used in our study, such as differences in the number and width of tubules depending on factors like the age of extracted teeth, could explain this discrepancy.

ClO_2_ has become a popular irrigation solution for root canal treatment in recent years. This is because it kills bacteria, dissolves organic tissue, and is less harmful to cells than NaOCl [8, 42]. Dang et al. [43] conducted an in vitro study, measuring the surface tension of 0.405% ClO_2_ at 26 °C using the pendant drop method, yielding a value of 59.28. We measured the surface tension value for 0.2% ClO_2_ in our current investigation to be 77.08. The disparity between the findings could stem from differences in temperature and concentration. No other study measuring both the surface tension of ClO_2_ and its contact angle on dentin surfaces was found in the literature; thus, our research contributes to the literature in this aspect.

HOCl is a potent oxidizing agent. In an aqueous solution, it dissociates into H + and OCl-, leading to the denaturation and aggregation of proteins [44]. HOCl also kills viruses by chlorinating them and creating chloramines and nitrogen-based radicals. These help break both single- and double-strand DNA, which makes viruses useless [45]. After reviewing the literature, several studies have investigated the use of HOCl as a surgical antiseptic and cavity disinfectant. Additionally, these studies investigated the impact of HOCl on enhancing the bonding of dentin bonding agents through the removal of proteins. Additionally, while there are articles exploring its role as a root canal antiseptic and pulp tissue solvent, as of now, there is no research evaluating its contact angle and surface tension properties [46–48]. Therefore, our study aims to contribute to the literature by investigating the contact angle and surface tension between HOCl and dentin surfaces.

In our study, although the surface tension values of NaOCl, ClO_2_, and HOCl were higher than those of distilled water, the contact angle with dentin surfaces was found to be lower than that with distilled water. It is observed that the high surface tension values of these solutions did not adversely affect the contact angle. Iglesias et al. [18] measured the contact angle of 2.5% NaOCl on root dentin, obtaining values of 44.7 in the cervical region and 44.63 apically. In our current study, the contact angle of 5.25% NaOCl was measured as 48.81. In an in vitro study conducted by Spano et al. [35], the surface tension and pH values of NaOCl before and after contact with pulp were examined, revealing a decrease in both values after contact with organic tissue. The researchers attributed this phenomenon to a variety of chemical reactions initiated by NaOCl upon contact with organic matter. Fatty acids react with sodium hydroxide to form soap and glycerol (saponification reaction); amino acids react with sodium hydroxide to form salt and water (neutralization reaction); and additionally, they react with HOCl to form chloramine and water. These reactions occur simultaneously and synergistically, leading to the liquefaction of organic tissue. It is also known that NaOCl deproteinizes hard tissues [49]. ClO_2_, a chlorine-based compound, is known for its organic tissue-dissolving effect [42]. In our study, the lower contact angles observed for NaOCl, ClO_2_, and HOCl compared to distilled water may be attributed to the decreased surface tensions resulting from their contact with the smear layer on dentin surfaces, as also suggested by Spano et al. [35], or to chemical reactions occurring at the interface [33].

Understanding both the surface tension and contact angles of irrigation solutions not only provides valuable data on the properties of these solutions but can also assist researchers in the development of advanced irrigation solutions for clinical applications. According to Giardino et al. (25), an irrigation solution with lower surface tension and/or dentin contact angle may remove microorganisms and debris in the root canal system more effectively. Therefore, the present study may guide the selection of clinically preferred solutions, and more successful treatment results may be achieved.

In our present study, although the surface tension values of EDTA, NaOCl, ClO_2_, BA, and HOCl were higher than those of distilled water, their contact angles with dentin surfaces were found to be lower than those of distilled water. Hence, it was observed that the high surface tension properties of the solutions did not adversely affect the contact angles of the root dentin. Therefore, it may be suggested that there might not be a direct linear relationship between surface tension and contact angles on different surfaces, and these values should be independently evaluated for each solution and surface type.

## Conclusion

- Based on the current study:
- It is crucial that the contact angle measurements of irrigation solutions be conducted on root canal dentin surfaces, rather than on any solid surface, to better reflect and facilitate clinical comparisons.
- There may not be a direct linear relationship between the surface tension of liquids and the contact angles on different surfaces, and these values may vary for each solution on different surfaces, thus necessitating independent evaluation.
- ClO_2_ and NaOCl are chlorinated compounds with similar properties; however, due to ClO_2_'s lower contact angle and surface tension with dentin compared to NaOCl, it may have the potential to be recommended instead of NaOCl.
- BA and EDTA are two effective solutions on smear layer removal. Since BA exhibits a better contact angle with dentin compared to EDTA, it could be recommended as an alternative to EDTA.

These conclusions have been drawn.

## Data Availability

The data presented in this study are available on reasonable request from the corresponding author.

## Abbreviations

NaOCl: Sodium hypochlorite
EDTA: Ethylenediaminetetraacetic acid
CHX: Chlorhexidine
BA: Boric acid
HOCl: Hypochlorous acid
ClO_2_: Chlorine dioxide
